# RSV Infection in Refugees and Asylum Seekers: A Systematic Review and Meta-Analysis

**DOI:** 10.3390/epidemiologia5020016

**Published:** 2024-05-27

**Authors:** Matteo Riccò, Silvia Corrado, Marco Bottazzoli, Federico Marchesi, Renata Gili, Francesco Paolo Bianchi, Emanuela Maria Frisicale, Stefano Guicciardi, Daniel Fiacchini, Silvio Tafuri

**Affiliations:** 1 AUSL–IRCCS di Reggio Emilia, Servizio di Prevenzione e Sicurezza Negli Ambienti di Lavoro (SPSAL), Local Health Unit of Reggio Emilia, 42122 Reggio Emilia, Italy; 2 ASST Rhodense, Dipartimento Della Donna e Area Materno-Infantile, UOC Pediatria, 20024 Garbagnate Milanese, Italy; scorrado@asst-rhodense.it; 3 Department of Otorhinolaryngology, APSS Trento, 38122 Trento, Italy; 4 Department of Medicine and Surgery, University of Parma, 43126 Parma, Italy; federico.marchesi@unipr.it; 5 Department of Prevention, Turin Local Health Authority, Via Silvio Pellico 19, 10125 Turin, Italy; 6 Health Prevention Department, Local Health Authority of Brindisi, 72100 Brindisi, Italy; frapabi@gmail.com; 7 Directorate General of Health Prevention, Ministry of Health, 00144 Rome, Italy; 8 Health Directorate, Local Health Authority of Bologna, 40127 Bologna, Italy; 9 Department of Biomedical and Neuromotor Sciences, University of Bologna, 40127 Bologna, Italy; 10 AST Ancona, Prevention Department, UOC Sorveglianza e Prevenzione Malattie Infettive e Cronico Degenerative, 60127 Ancona, Italy; 11 Department of Interdisciplinary Medicine, Aldo Moro University of Bari, 70121 Bari, Italy

**Keywords:** RSV, viral pneumonia, differential diagnosis, homelessness, influenza, refugee campa, SARS-CoV-2

## Abstract

Respiratory diseases, including respiratory syncytial virus (RSV) infections, are common reasons for seeking healthcare among refugees and asylum seekers. A systematic review with meta-analysis was designed to appraise all the available evidence on RSV infections among individuals in refugee camps. Three medical databases (PubMed, Embase, and Scopus) as well as the preprint repository medRxiv.org were searched for eligible observational studies, and the collected cases were pooled in a random-effects meta-analysis model. Heterogeneity was assessed using the I^2^ statistics. Funnel plots and a regression analysis were calculated for analyzing reporting bias. Eventually, six studies were retrieved from three areas (Bangladesh, Thailand, and Kenya), with pooled estimates of 129.704 cases per 1000 samples (95% CI 66.393 to 237.986) for RSV compared to 110.287 per 1000 people for influenza A (95% CI 73.186 to 162.889), 136.398 cases per 1000 people (95% CI 84.510 to 212.741) for human adenovirus (HAdV), 69.553 per 1000 people (95% CI 49.802 to 96.343) for parainfluenzavirus (PIFV), and 60.338 per 1000 people (95% CI 31.933 to 111.109) for human metapneumovirus (hMPV). Using influenza A as a reference group, the risk for a positive specimen was greater for RSV (relative risk [RR] 1.514, 95% CI 1.396 to 1.641) and HAdV (RR 1.984, 95% CI 1.834 to 2.146) and lower for influenza B (RR 0.276, 95% CI: 0.239 to 0.319), PIFV (RR: 0.889, 95% CI 0.806 to 0.981), and hMPV (RR 0.594, 95% CI 0.534 to 0.662). In summary, high rates of RSV infections were documented among individuals sheltered in refugee camps, stressing the importance of specifically designed preventive strategies.

## 1. Introduction

According to the United Nations High Commissioner for Refugees (UNHCR), the UN agency working to protect refugees, forcibly displaced communities, and stateless people, a total of 108.4 million people worldwide were forcibly displaced in 2022, with an increase of around 19 million people compared to 2021. This was mostly due to refugees from Ukraine fleeing the international armed conflict in their country. Of them, around 35.3 million people were represented by refugees, including 29 million people under the direct UNHCR mandate and 5.4 million asylum seekers. Most refugees, up to 76%, are hosted by low- (16%) and middle-income countries (59%) [1].

Refugees are usually considered at particularly high risk for infectious diseases for a series of reasons, including the difficult living conditions with overcrowding, poor water and sanitation conditions; the limited access to timely diagnosis; and the missed opportunities for prevention vaccination and treatment [2,3,4,5,6,7]. Because of the aforementioned conditions, epidemics among refugees are also associated with higher morbidity and case fatality ratios, stressing both the precarious conditions of the displacement and the challenges in accessing care [2,8]. Even though, outbreaks of gastro-intestinal diseases [9] as well as measles [10,11], varicella [12,13], and protozoan infections [14,15] have been extensively reported in these population groups, and acute respiratory infections (ARI) are usually acknowledged as the most common reasons for seeking medical advice among refugees and displaced people [16,17,18,19,20]. Among the whole of ARIs, infections due to respiratory syncytial virus (RSV) appear of particular interest for healthcare professionals for a series of reasons. RSV is an enveloped and pleomorphic virus of medium size (120–300 nm diameter; genus *Orthopneumovirus*, family Pneumoviridae) belonging to the mononegavirales order that includes viruses with a single stranded negative sense RNA of around 15 to 16 kilobases [21,22,23,24]. RSV is usually acknowledged as a highly contagious pathogen [25,26]. Before the inception of the COVID-19 pandemic, RSV was acknowledged as the single most common viral cause of lower respiratory tract infections (LRTI) [23]. In fact, available global estimates hint at around 33 million cases of ARI and LRTI in infants aged 5 years or less every year [22,23,27], with high hospitalization rates [28,29,30,31,32] leading to around 3.5 million hospital admissions [21,23]. When dealing with the global burden of RSV and with RSV-associated direct and indirect costs, it is important to stress that RSV also affects older individuals [33,34,35,36]. In the European Union alone, it causes around 160,000 hospitalizations annually in adults aged ≥ 18 years, with 92% of cases involving adults aged ≥ 65 years [37], for a corresponding hospitalization rate of around 157 per 100,000 [38]; however, these figures are reasonably underestimate because of the low rate of testing in adults elderly. Even though COVID-19 pandemic was associated with a substantial decrease in global rates for RSV-associated hospitalization (−79.7% in high income countries, −13.8% in upper-middle-income countries), post-pandemic seasons were associated with a sustained rebound of incidence estimates, with increased healthcare-associated costs [37,39,40]. On the other hand, a series of recent reports hint toward the relatively high occurrence of RSV infections among individuals hosted in urban shelters for homeless people [20,41,42], a population sharing several features with refugees and displaced people [4,15,43,44,45,46].

Nevertheless, the actual burden of RSV among refugees, asylum seekers, and displaced people may be potentially greater than that identified among homeless people from urban shelters. In fact, studies on homeless people usually oversample individuals of male gender in age groups ranging from 20 to 50 years, while reports from refugee camps are characterized by a high prevalence of subjects from either low or high age groups. For example, in a recent report from Siddik et al. [47], around 59% of sampled individuals were less than 5 years; however, in the previous report of Ahmed et al. [48], only 18.0% of 6264 specimens were collected from individuals older than 5 years. According to available estimates, the overwhelming majority of children is usually infected by RSV before the 2nd year of age [21,29,30], and the majority of complications leading to the eventual hospitalization and admission to intensive care units (ICUs) occur in otherwise healthy children [49,50,51,52]. Until recently, very few effective mitigation strategies for RSV were ultimately available, while the recent licensing of maternal and adult vaccines and cost-effective preventive monoclonal antibodies (mAb) [25,53,54,55,56,57,58,59] could lead to a series of evidence-based public health interventions to mitigate the impact of this pathogen, particularly in high-risk settings.

While relatively little is known about the epidemiology of RSV in refugee camps, understanding and addressing this specific topic could be of paramount relevance in ensuring the safety of refugee communities and preventing the further escalation of respiratory infections in already challenging circumstances. Consequently, we designed the present systematic review and meta-analysis in order to answer two main research questions: (1) What is the occurrence of RSV infections in refugee camps? and (2) Is the occurrence of RSV comparable to that of other respiratory pathogens?

## 2. Materials and Methods

### 2.1. Research Concept

This systematic review was designed in accordance with the guidelines from the Preferred Reporting Items for Systematic Reviews and Meta-Analysis (PRISMA) statement [60,61] (see Supplementary Table S1) and was preliminary registered on the PROSPERO database, an international prospective registry of systematic reviews (CRD42023475548). The objective of the study was defined based on the PECO strategy (i.e., Patient/Population/Problem; Exposure; Control/Comparator; Outcome) [62,63]. More specifically, we were interested in the following research question: among adults and children (P) sheltered in for refugee camps (E) is the occurrence (i.e., prevalence and/or incidence) of influenza-like illnesses (ILIs) or severe acute respiratory illnesses (SARIs) associated with RSV infections (O) comparable to the estimates for other respiratory pathogens, including influenza A, influenza B, adenovirus (HAdV), para-influenza virus (PIFV), and human metapneumovirus (hMPV) (C)?

### 2.2. Research Strategy

Three databases (i.e., PubMed, using of Medical Subject Heading [MeSH] terms; EMBASE; and Scopus) and the preprint repository medRxiv were searched using a specifically designed research strategy from inception up to 30 March 2024; the corresponding search scripts and text-wide terms are reported in Table A1.

### 2.3. Screening

All types of observational studies were potentially eligible, irrespective of the modality and time of publication (e.g., peer-reviewed articles, preprint, conference abstract, etc.). The condition of interest was represented by the occurrence of RSV infection, only documented by real-time quantitative polymerase chain reaction (RT-qPCR) assays. Regarding the context, we only considered studies performed in refugee camps, in any time and calendar period. Population consisted of all sampled individuals, irrespective of their underlying conditions and numerosity. For the aims of this review, the following definition of refugee camp was applied: “any temporary facility built to provide immediate protection and assistance to people who have been forced to flee their homes due to war, persecution or violence” (https://www.unrefugees.org/refugee-facts/camps/, accessed on 12 April 2024). On the contrary, studies performed with refugees dwelling in urban settings, including urban shelters as well as on refugees and asylum seekers living in homeless settings (i.e., streets, open spaces or cars, or severely inadequate and insecure housing), were not included [64,65]. We deliberately ruled out these specific population groups because of the presumptively high proportion of male individuals from urban shelters, with reduced proportions of children and adolescents compared to those usually reported by refugee camps [66].

The following exclusion criteria were then applied:

(1) The full text was not available either through online repositories or through inter-library loan or its main text was written in a language different from those understood by the Study Authors (English, Italian, German, French, Spanish, or Portuguese);
(2) The study was designed as a case report and/or a case series;
(3) The study was designed as a modeling and/or pharmacoeconomic study or a review/systematic review or was a study lacking original data;
(4) The study reported on respiratory pathogens other than RSV;
(5) Redundant publication;
(6) Geographical and time settings were not provided;
(7) Sampling approach as well as inclusion/exclusion criteria for sample collection were not provided;
(8) The study did not provide the total number of sampled individuals; and
(9) Laboratory diagnosis of respiratory infections was performed on methods other than RT-qPCR (e.g., clinical features, imaging, seroprevalence studies, etc.).

All entries consistent with inclusion criteria and not excluded based on the exclusion criteria were subject to title screening; if the title was relevant to the research questions, the abstract was then analyzed [60,67]. All the entries that were considered consistent with the aims of research questions underwent full-text screening. Two investigators (SC, FM) independently rated each entry. Cases of disagreements were tentatively discussed; when the consensus was not reached, the chief investigator (MR) was consulted.

### 2.4. Summary of Retrieved Data

Abstracted data included the following:

(a) Settings of the study: Time of the study and/or observation period(s), country (region), and timeframe. If a certain study provided data on distinctive timeframes, estimates were separately reported and analyzed as distinctive series.
(b) Total number of refugees potentially included in the estimate(s) and their demographic data (age, gender);
(c) Number of collected samples (total);
(d) Number of reported cases of ILI and/or SARI and/or pneumonia; and
(e) Number of samples having a positive RT-qPCR diagnosis for the following respiratory pathogens: RSV, influenza A and B, PIFV, HAdV, or hMTP.

### 2.5. Risk of Bias Analysis

Flaws in research practices ultimately bias the study design and resulting estimates [68,69,70], potentially impairing the validity of the collected quantitative evidence. To cope with the underlying risk of bias (ROB) from retrieved studies, the ROB tool from the Health Assessment and Translation group of the National Institute of Environmental Health Sciences (formerly Office of Health Assessment and Translation (OHAT)) was therefore implemented [70,71]. OHAT ROB is designed to assess the internal validity of individual studies (i.e., whether the design and conduct of the study compromised the credibility of the link between exposure and outcome) by weighting a series of potential sources of bias: participant selection (D1), confounding factors (D2), attrition/exclusion (D3), detection (D4), selective reporting (D5), as well as other sources of bias (D6). All sources of bias are rated as “definitely low,” “probably low”, “probably high”, or “definitely high” regarding the likelihood that they compromise the association between exposure and reported outcome. OHAT ROB does not provide an overall rating for each study, and it was preferred over other similarly designed instruments as its framework recommends that even studies reasonably affected by substantial ROB would be retained into the pooled analyses in order to avoid the underestimation of the health effects from the considered exposure [71].

### 2.6. Data Analysis

As a preliminary step, prevalence rates for RSV and all other respiratory pathogens were calculated as the number of positive specimens over the whole number of collected samples. All estimates were initially reported as numbers per 1000 specimens.

Because of its global disease burden, the prevalence estimate for influenza A was arbitrarily assumed as the reference group, and risk ratios (RRs) with their corresponding 95% confidence intervals (95% CIs) were accordingly calculated using bivariate analysis for RSV and all other respiratory pathogens. The RRs for all respiratory pathogens were similarly calculated by arbitrarily assuming the prevalence of positive samples in cases of ILI vs. cases of SARI/pneumonia as the reference categories.

Pooled prevalence estimates were calculated using a random effect model (REM) meta-analysis of retrieved studies. Data were reported as estimates for all retrieved studies and by geographic area. We deliberately preferred REM over a fixed effect model as it is considered more effective in dealing with the study heterogeneity and the presumptive variation in study outcomes [72,73].

The inconsistency of effect between the studies was defined as the percentage of total variation likely due to heterogeneity rather than chance [68] and quantified using I^2^ statistic calculations. I^2^ estimates were then classified as follows: 0 to 25%, low heterogeneity; 26% to 50%, moderate heterogeneity; ≥50%, substantial heterogeneity. The 95% CIs of I^2^ estimates were provided to cope with the potential small size of the meta-analyses [68].

To investigate the sources of uncertainty across the includes studies, sensitivity ana-lysis evaluated the effect of each study on the pooled estimates through the exclusion of one study at a time.

Potential publication bias was ascertained through calculation of contour-enhanced funnel plots and using the Egger’s test [60,74]. Radial plots were eventually calculated in order to assess small study bias.

All calculations were performed in R (version 4.3.2) [75] and Rstudio (version 2023.06.0 Build 421; Rstudio, PBC; Boston, USA) software using the packages meta (version 6.5-0) and fmsb (version 0.7.5). The Prisma2020 flow diagram was designed using the PRISMA2020 package [76].

## 3. Results

### 3.1. Descriptive Analysis

**Figure 1:**
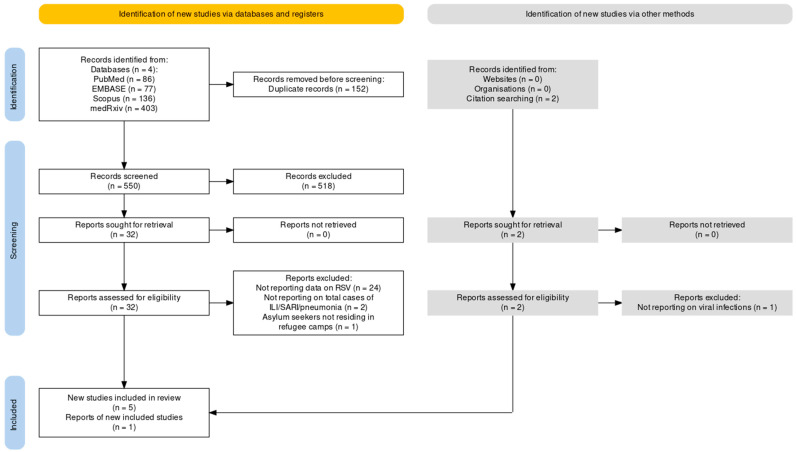
Flow chart of included studies [5,7,45,47,48,77].

As the flowchart in Figure 1 shows (Appendix A, Table A1), a total of 702 entries were identified from searched databases. More precisely, 403 (57.41%) studies were identified from the preprint repository of medRxiv, 136 (19.37%) from Scopus, 86 (12.25%) from PubMed, and 77 from Embase (10.97%).

After the removal of duplicated studies (152, 21.65%), the titles and abstracts of 550 articles were screened, and a total of 518 records were removed (73.79% of the initial sample). In total, 32 entries were ultimately assessed for their eligibility with full-text review (4.56%). Of those reports, 27 were then removed from the analyses based on not reporting data on RSV (24, 3.42%) or not providing the total number of incident cases of ARI, ILI, or SARI/pneumonia (2, 0.28%), and a single study was removed based on providing data from asylum seekers not living in shelters and/or specifically designed camps [78]. Five retrieved studies [5,7,47,48,77] were ultimately identified, and an additional entry [45] was identified using citation screening. Detailed characteristics of the studies included in qualitative and quantitative analysis are provided in Table 1.

### 3.2. Characteristics of Prevalence Studies

**Figure 2:**
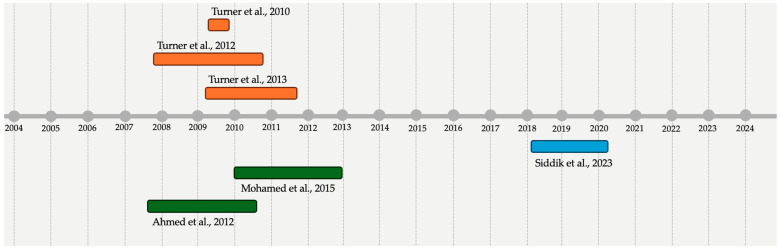
Distribution of included studies over time (♦ = Thailand; ♦ = Kenya; ♦ = Bangladesh) [5,7,45,47,48,77].

The six studies reported on data retrieved from three distinctive areas with study dates ranging from November 2007 to March 2020, and substantial overlap of the inquired areas was noted (Figure 2). Overall, 2 studies were from Kenya (2007–2010) and included refugees sheltered in the camps of Hagadera, with a potential reference population ranging from 305,000 [48] and 122,068 refugee people [77]. Three studies documented viral infections at the Mae La refugee camp in Thailand (timeframe November 2007 to September 2011) [5,7,45], with a population of around 45,000 refugee people at the time of the studies (2007 to 2010). Eventually, 1 study reported on the refugee camp of Cox’s Bazaar in Bangladesh [47] from March 2018 to March 2020, and according to the official estimates from the UNHCR, during that timeframe, the inquired area hosted a total of 598,545 refugee people.

As Table 1 summarizes, the sampling strategy was heterogenous. The oldest study from Turner et al. [45] reported on all incident cases of ILI or pneumonia, while both reports from Kenya included incident cases affected by either ILI or SARI [48,77]. In addition, Mohamed et al. also reported data on documented ARI cases in the parent refugee population [77]. On the contrary, Siddik et al. did not dichotomize their report based the severity of reported infections and included in their estimates all incident ARI cases [47]. Both remaining reports from the Mae La camp only included data on pneumonia [5,7]. Although the report by Turner et al., 2012 was designed as a cohort study, including data from 965 children followed from birth until 2 years of life [5], the other report from Turner et al., 2013 included all incident pneumonia cases, all of which were sampled for both viral and bacterial respiratory pathogens [7].

Overall, a total of 9633 people were included in the pooled estimates. The largest number of sampled patients was provided by the study from Ahmed et al. (6647; 69.00%) [48]; followed by the cohort study by Turner et al., 2012 (955, 9.91%) [5,7]; the study from Turner et al., 2013 on incident pneumonia cases (698, 7.25%); the report on Cox’s Bazar from Siddik et al. (538, 5.58%) [47]; the study from Mohamed et al. (471, 4.89%); and the study from Turner et al., 2010 (324, 3.36%) [45].

Similarly, the largest number of samples was provided by the study from Ahmed et al. (6254; 67.22%) [48]; followed by Turner et al., 2012 (1085; 11.64%) [5]; Turner et al., 2013 (708, 7.60%) [7]; Siddik et al. (538, 5.77%) [47]; Mohamed et al. (419, 4.50%) [77]; and Turner et al., 2010 (305, 3.27%). The pooled sample included a total of 2086 samples of ILI tested for RSV, influenza A, influenza B, and hMPV, while 2015 specimens were tested for PIFV and HAdV. Moreover, a total of 6645 cases of SARI/pneumonia were tested for RSV; 5560 were sampled for influenza A, influenza B, and hMPV; 5326 for HAdV; and 4618 for PIFV.

Overall (Table 2), 15.18% of all samples was positive for RSV, with point estimates ranging from 3.82% in Mohamed et al. and 33.36% in Turner et al., 2012. Regarding the other sampled pathogens, the highest occurrence was associated with HAdV (19.90%), with point estimates ranging from 6.32% in Siddik et al. [47] to 21.73% in Ahmed et al. [48] Nonetheless, 10.03% of samples were positive for influenza A (range 5.95% to 22.04%), 8.92% for PIFV (range 5.39% to 9.43%), and 5.96% for hMPV (range 3.34% to 21.38%). The lowest occurrence was noted for influenza B (2.88%, range 0 to 8.11%).

In other words, the large majority of collected data was drawn from Kenya (73.89% of patients and 71.72% of samples), followed by Thailand (20.52% of patients and 22.51% of samples), and the single study from Siddik et al. reporting on Bangladesh, representing 5.58% of assessed refugee people and 5.77% of retrieved samples.

As shown in Table 3, the prevalence of RSV was therefore the highest in Kenya (30.01%), followed by Thailand (12.18%), with the lowest rates noted in Bangladesh (4.83%). On the contrary, the highest rates for HAdV, influenza A, and hMPV were identified in samples from Thailand (21.11%, 10.53%, and 6.27%, respectively), followed by Kenya (18.79%, 8.19%, and 4.66%), and Bangladesh (6.32%, 5.95%, and 3.72%). On the contrary, the highest rates for influenza B were identified in Kenya (8.19%), followed by Bangladesh (4.28%), and Thailand (2.79%).

As studies from Kenya did not track PIFV, available data were drawn only from Thailand studies (prevalence 9.20%) and from the report on Cox’s Bazar (5.39%).

### 3.3. Univariate Analysis

**Figure 3:**
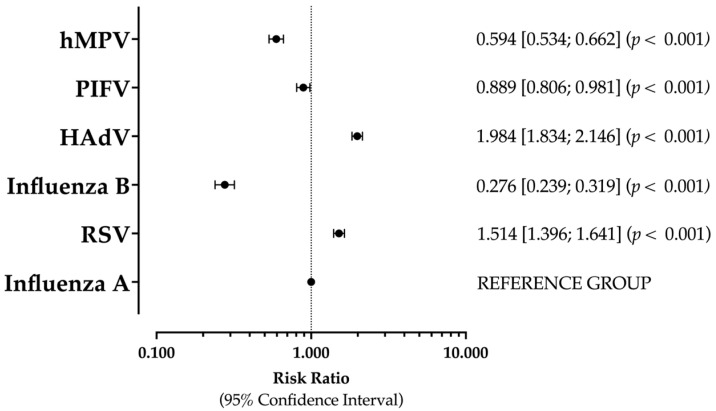
Risk ratios (RRs) and their respective 95% confidence intervals (95% CIs) for positive status of the sampled respiratory pathogens. In the analyses, prevalence rate of influenza A was considered as the reference estimate. Note: RSV = respiratory syncytial virus; HAdV = human adenovirus; PIFV = parainfluenza virus; hMPV = human metapneumovirus [5,7,45,47,48,77].

As Figure 3 shows, when prevalence rate for influenza A was considered the reference estimate, both RSV and HAdV were associated with increased risks for a positive specimen (RR 1.514, 95% CI 1.396 to 1.641; RR 1.984, 95% CI 1.834 to 2.146). On the contrary, the risk for a positive specimen was significantly reduced for remaining pathogens, including influenza B (RR 0.276, 95% CI 0.239 to 0.319), PIFV (RR 0.889, 95% CI 0.806 to 0.981), and hMPV (RR 0.594, 95% CI 0.534 to 0.662).

**Figure 4:**
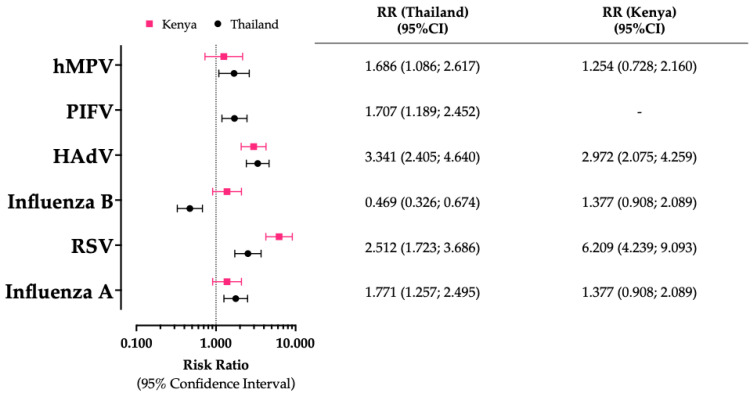
Risk ratios (RRs) and their respective 95% confidence intervals (95% CIs) for positive status of sampled respiratory pathogens in studies from Thailand and Kenya compared to estimates from the study from Siddik et al. on Cox’s Bazar, Bangladesh (2023). Note: RSV = respiratory syncytial virus; HAdV = human adenovirus; PIFV = parainfluenza virus; hMPV = human metapneumovirus [5,7,45,47,48,77].

As summarized in Figure 4, the risk for a positive specimen was heterogenous not only based on the specifically assessed pathogen, but also by geographical area. For instance, when estimates from Bangladesh were arbitrarily taken into account as the reference group [47], the RR for reporting a RSV-positive specimen was not only significantly greater in Kenya (RR 6.209, 95% CI 4.249 to 9.093) than the reference category but also Thailand (RR 2.512, 95% CI 1.723 to 3.686). Furthermore, occurrence of HAdV was significantly higher in Thailand and Kenya than in Bangladesh, but eventual estimates were not substantially different (RR 3.341, 95% CI 2.405 to 4.460; RR 2.972, 95% CI 2.075 to 4.259). Moreover, the occurrence of influenza A and hMPV was significantly higher in Thailand (RR 1.771, 95% CI 1.257 to 2.495; RR 1.686, 95% CI 1.086 to 2.617, respectively) than in the reference group and among refugees from Kenya (RR 1.377, 95% CI 0.908 to 2.089; RR 1.254, 95% CI 0.728 to 2.160, respectively). The occurrence of influenza B was significantly lower among refugees from Thailand (RR 0.469, 95% CI 0.326 to 0.674) than in the reference population of Bangladesh, where estimates were not significantly different from those collected in Kenyan studies (RR 1.377, 95% CI 0.908 to 2.089). As PIFV was not sampled in studies from Ahmed et al. [48] and Mohamed et al. [77], cumulative comparisons were performed only with studies on the Mae La refugee camp, where an increased occurrence of this pathogen compared to Bangladesh was eventually documented (RR 1.707, 95% CI 1.189 to 2.452).

**Figure 5:**
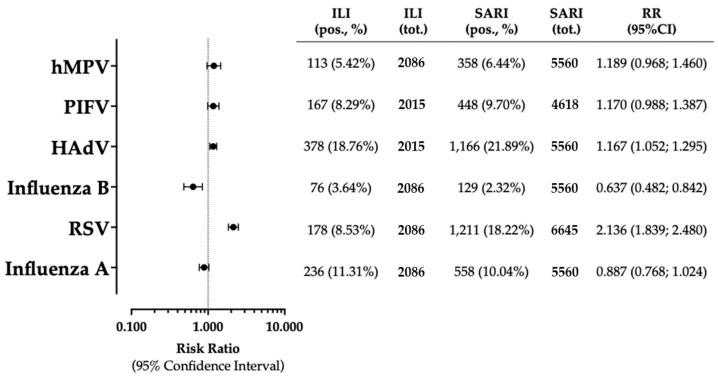
Risk ratios (RRs) and their respective 95% confidence intervals (95% CIs) for positive status of sampled respiratory pathogens among cases of SARI/pneumonia compared to those of ILIs. Note: RSV = respiratory syncytial virus; HAdV = human adenovirus; PIFV = parainfluenza virus; hMPV = human metapneumovirus [5,7,45,47,48,77].

In Figure 5, the risk for a positive specimen was compared between reported ILIs and documented SARI/pneumonia cases. In 8.53% of 2086 ILIs sampled for RSV, a pathogen was eventually identified, compared to 18.22% of 6645 sampled SARIs. In other words, among the assessed refugees, the occurrence of RSV-positive specimens was significantly greater in SARI/pneumonia cases than among ILIs (RR 2.136, 95% CI 1.839 to 2.480).

Similarly, the occurrence of HAdV-positive samples was more frequently reported among SARI cases than among ILI cases (21.89% vs. 18.76%; RR 1.167, 95% CI 1.052 to 1.295), while influenza B was more frequently reported among ILI cases than among SARI cases (3.64% vs. 2.32%, respectively; RR 0.637, 95% CI 0.482 to 0.842). Regarding the remaining pathogens (i.e., influenza A, PIFV, hMPV), no significant differences were documented.

### 3.4. Risk of Bias Assessment

**Figure 6:**
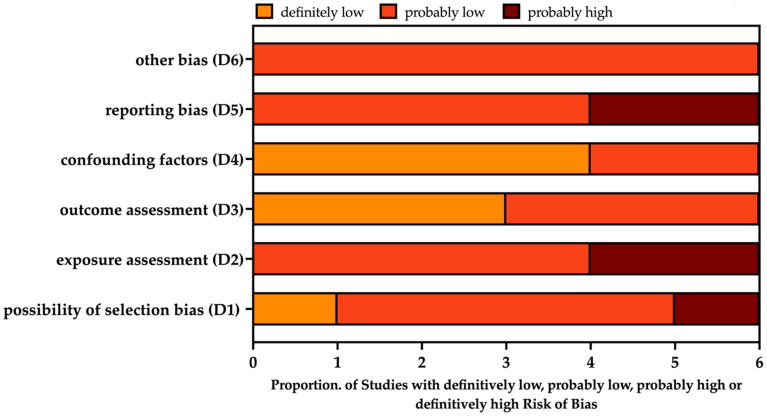
Summary of the risk of bias (ROB) estimates for observational studies [71,79]. Analyses were performed according to the National Toxicology Program (NTP)’s Office of Health Assessment and Translation (OHAT) handbook and respective risk of bias (ROB) tool [5,7,45,47,48,77].

Overall, the individual risk of bias was probably low or definitively low for most of the assessed domains (Figure 6).

Focusing on individual reports (Table 4), the study by Siddik et al. [47] was possibly affected by some degree of selection and exposure assessment bias as the authors included all incident cases of ARI and the proportion of ILI/SARI and/or pneumonia cases was not provided. Similarly, the study by Turner et al. 2012 [5] focused on a cohort of 955 children born in the Mae La camp. As some of them relocated during the study period, we cannot rule out that some exposures were not associated with living in the refugee camp.

Moreover, demographic data on these subjects were only provided for cases with a positive RSV specimen, impairing a more comprehensive appraisal of recruited patients, hinting at probable reporting bias. A probable high risk of reporting bias was also associated with report by Ahmed et al., as the authors were unable to collect data on all eligible individuals, and particularly cases of ILI were likely underrepresented.

### 3.5. Meta Analysis

**Figure A1:**
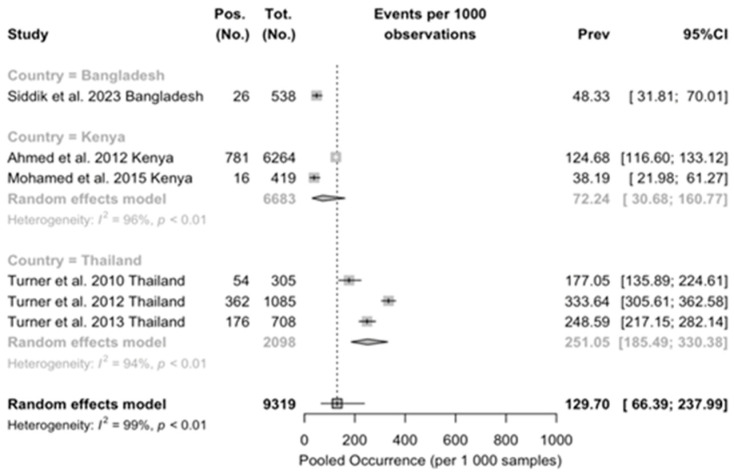
Pooled occurrence of RSV-positive samples in retrieved studies. A pooled rate of 129.704 per 1000 specimens (95% confidence interval [95% CI] 66.393 to 237.986) was eventually identified. Occurrence was highest for studies performed in Thailand (251.046 per 1000 specimens, 95% CI 185.485 to 330.379), followed by Kenya (72.241 per 1000 specimens, 95% CI 30.680 to 160.765), and Bangladesh (48.327 per 1000 specimens, 95% CI 33.110 to 70.031). All estimates were affected by substantial heterogeneity, with I^2^ for individual subgroups exceeding 95% [5,7,45,47,48,77].

**Figure A2:**
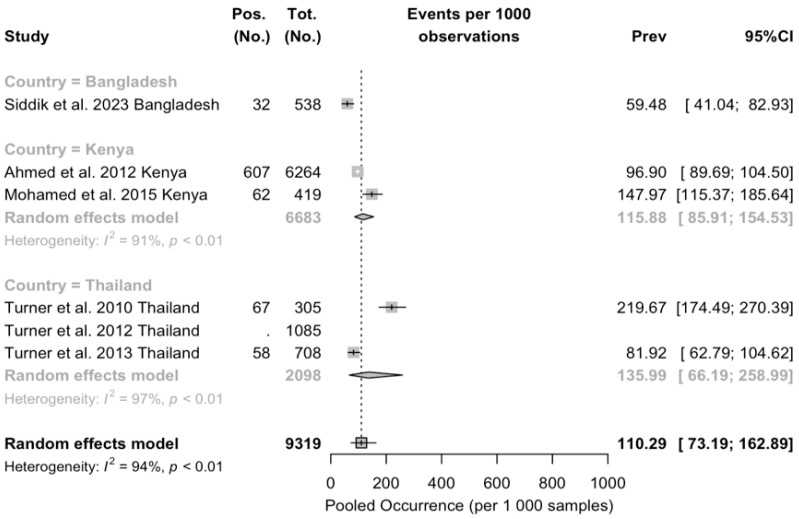
Pooled occurrence of influenza A-positive samples in retrieved studies. A pooled rate of 110.287 per 1000 specimens (95% confidence interval [95% CI] 73.186 to 162.889) was eventually identified. Occurrence was highest for studies performed in Thailand (135.995 per 1000 specimens, 95% CI 66.194 to 258.987), followed by Kenya (115.877 per 1000 specimens, 95% CI 85.192 to 154.526), and Bangladesh (59.480 per 1000 specimens, 95% CI 42.368 to 82.903). All estimates were affected by substantial heterogeneity, with I^2^ for individual subgroups exceeding 90% [5,7,45,47,48,77].

**Figure A3:**
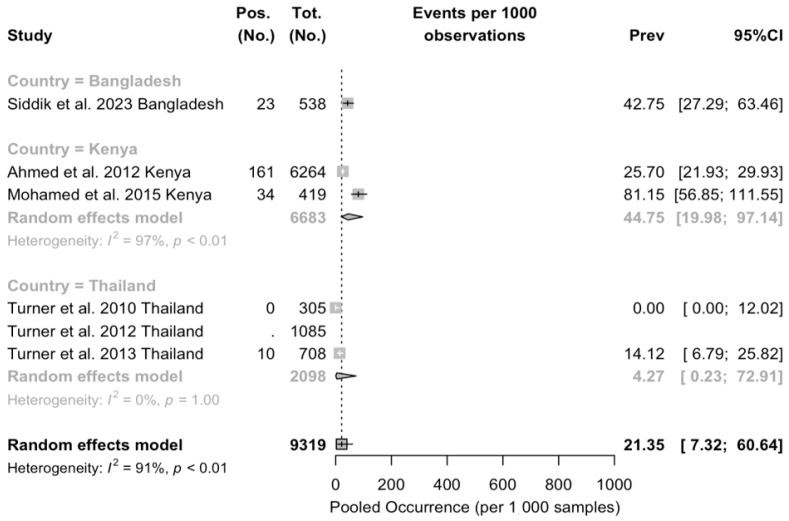
Pooled occurrence of influenza B-positive samples in retrieved studies. A pooled rate of 21.351 per 1000 specimens (95% confidence interval [95% CI] 7.319 to 60.639) was eventually identified. Occurrence was highest for studies performed in Kenya (44.745 per 1000 specimens, 95% CI 19.985 to 97.142), followed by Bangladesh (42.751 per 1000 specimens, 95% CI 28.571 to 63.509), and Thailand (4.271 per 1000 specimens, 95% CI 0.234 to 72.915). Pooled estimates were affected by substantial heterogeneity, with the corresponding I^2^ equal to 91.5% (95% CI 83.1 to 95.7) for the whole sample, I^2^ = 97.4% for Kenya, and not reported for Thailand-based studies [5,7,45,47,48,77].

**Figure A4:**
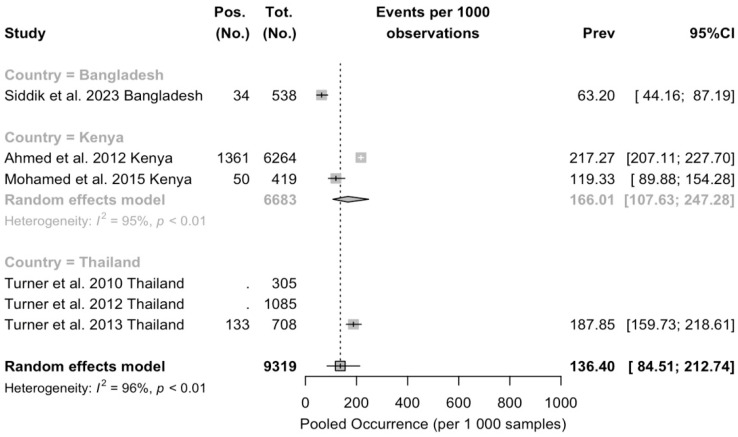
Pooled occurrence of adenovirus-positive samples in retrieved studies. A pooled rate of 136.398 per 1000 specimens (95% confidence interval [95% CI] 84.510 to 212.741) was eventually identified. Occurrence was highest for studies performed in Thailand (187.853 per 1000 specimens, 95% CI 160.757 to 218.328), followed by Kenya (166.009 per 1000 specimens, 95% CI 107.631 to 247.258), and Bangladesh (63.197 per 1000 specimens, 95% CI 45.499 to 87.151). Pooled estimates were affected by substantial heterogeneity, with a corresponding I^2^ value equal to 96.4% (95% CI 93.3 to 98.0) for the whole sample. Heterogeneity was calculated only for Kenya-based studies (I^2^ = 95.4%), as other subgroups only included one study [5,7,45,47,48,77].

**Figure A5:**
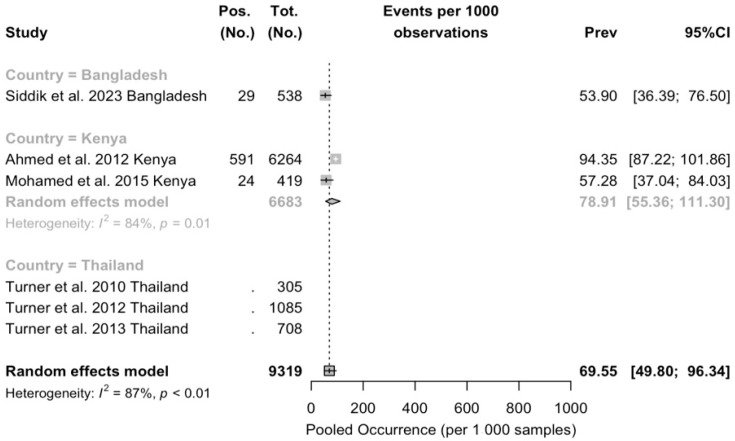
Pooled occurrence of parainfluenza virus-positive samples in retrieved studies. A pooled rate of 69.553 per 1000 specimens (95% confidence interval [95% CI] 49.802 to 96.343) was eventually identified. Pooled occurrence was calculated only for studies based in Kenya (78.910 per 1000 specimens, 95% CI 55.359 to 111.299) and compared to the single estimate from Bangladesh (53.903 per 1000 specimens, 95% CI 37.712 to 76.494). Pooled estimates were affected by substantial heterogeneity, with the corresponding I^2^ equal to 86.8% (95% CI 62.2 to 95.4) for the whole sample. Heterogeneity was calculated only for Kenya-based studies (I^2^ = 84.2%) [5,7,45,47,48,77].

**Figure A6:**
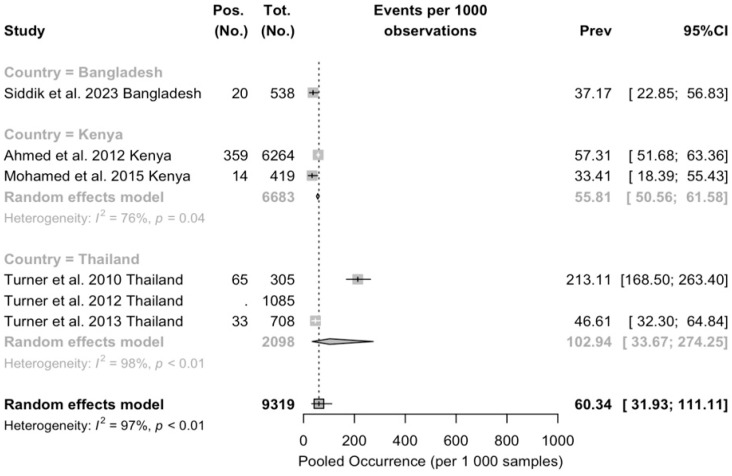
Pooled occurrence of human metapneumovirus-positive samples in retrieved studies. A pooled rate of 60.338 per 1000 specimens (95% confidence interval [95% CI] 31.933 to 111.109) was eventually identified. Occurrence was highest for studies performed in Thailand (102.940 per 1000 specimens, 95% CI 33.674 to 274.249), followed by Kenya (55.813 per 1000 specimens, 95% CI 50.558 to 61.579), and Bangladesh (37.175 per 1000 specimens, 95% CI 24.106 to 56.915). Pooled estimates were affected by substantial heterogeneity, with the corresponding I^2^ equal to 96.6% (95% CI 94.3 to 98.0) for the whole sample. Heterogeneity was calculated substantial also for subgroups of Kenya-based studies (I^2^ = 75.9%) and for Thailand-based studies (I^2^ = 98.2%) [5,7,45,47,48,77].

Pooled prevalence rates were reported as documented episode per 1000 specimens and were estimated using a REM meta-analysis. Corresponding estimates are summarized in Table 5, while individual Forrest plots are provided as Figure A1, Figure A2, Figure A3, Figure A4, Figure A5 and Figure A6.

More precisely, the greatest pooled estimate was identified for HAdV (136.398 per 1000 specimens, 95% CI 84.510 to 212.741), followed by RSV (129.704 per 1000 specimens, 95% CI 66.393 to 237.986), influenza A (110.287 per 1000 specimens, 95% CI 73.186 to 162.889), PIFV (69.553 per 1000 specimens, 95% CI 49.802 to 96.343), hMPV (60.338, 95% CI 31.933 to 111.109), and eventually influenza B (21.351 per 1000 specimens, 7.319 to 60.639). Overall, all the aforementioned estimates were affected by substantial heterogeneity. Specifically, I^2^ estimates were consistently >80%, and corresponding 95% CIs were characterized by lower limits exceeding the cut-off for substantial heterogeneity (i.e., 50%). Subgroup analysis showed that RSV occurrence was highest for studies performed in Thailand (251.046 per 1000 specimens, 95% CI 185.485 to 330.379), followed by Kenya (72.241 per 1000 specimens, 95% CI 30.680 to 160.765), and Bangladesh (48.327 per 1000 specimens, 95% CI 33.110 to 70.031). A similar trend was associated with estimates for influenza A (135.995 per 1000 specimens, 95% CI 66.194 to 258.987 for Thailand; 115.877 per 1000 specimens, 95% CI 85.192 to 154.526 for Kenya; and 59.480 per 1000 specimens, 95% CI 42.368 to 82.903 for Bangladesh), HAdV (187.853 per 1000 specimens, 95% CI 160.757 to 218.328 for Thailand; 166.009 per 1000 specimens, 95% CI 107.631 to 247.258 for Kenya; and 63.197 per 1000 specimens, 95% CI 45.499 to 87.151 for Bangladesh), and hMPV (102.940 per 1000 specimens, 95% CI 33.674 to 274.249 for Thailand; followed by 55.813 per 1000 specimens, 95% CI 50.558 to 61.579 for Kenya; and 37.175 per 1000 specimens, 95% CI 24.106 to 56.915 for Bangladesh). A different trend was identified for influenza B, with highest occurrence noted in studies performed in Kenya (44.745 per 1000 specimens, 95% CI 19.985 to 97.142) followed by Bangladesh (42.751 per 1000 specimens, 95% CI 28.571 to 63.509), and the lowest pooled rates were noted in Thailand (4.271 per 1000 specimens, 95% CI 0.234 to 72.915). All estimates were affected by substantial heterogeneity, with I^2^ for individual subgroups, where calculated, exceeding the cut-off value of 50% and mostly >90%.

### 3.6. Sensitivity Analysis

**Figure A7:**
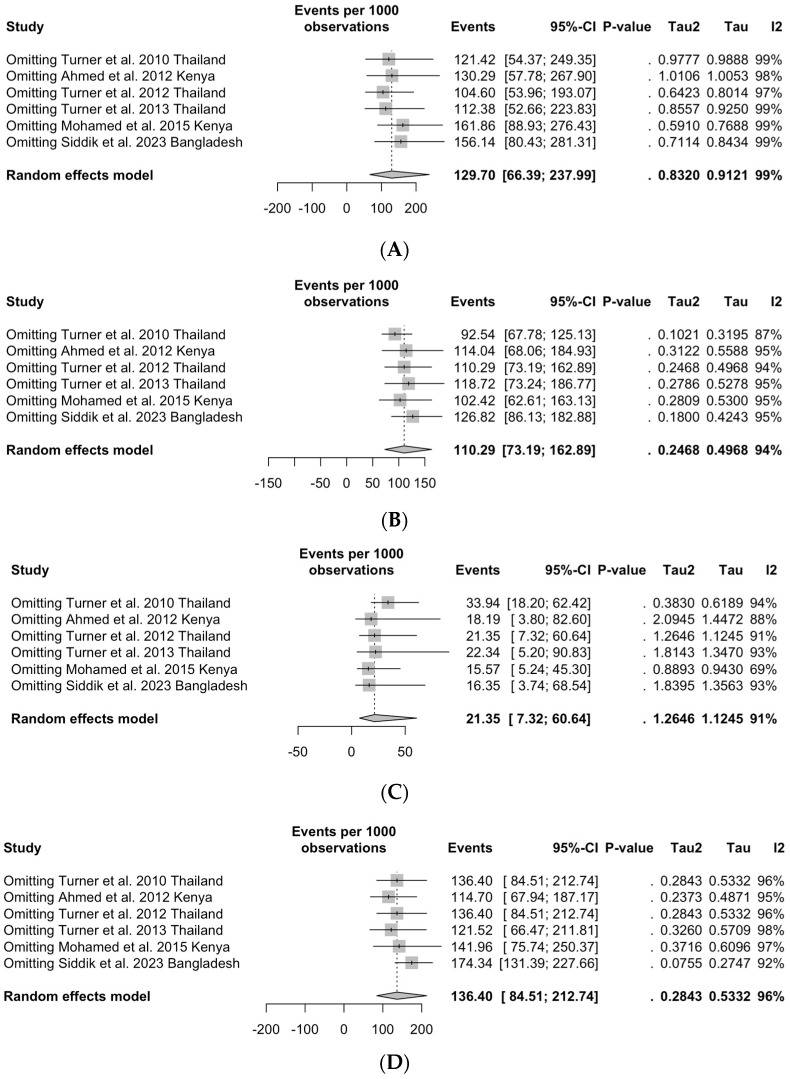

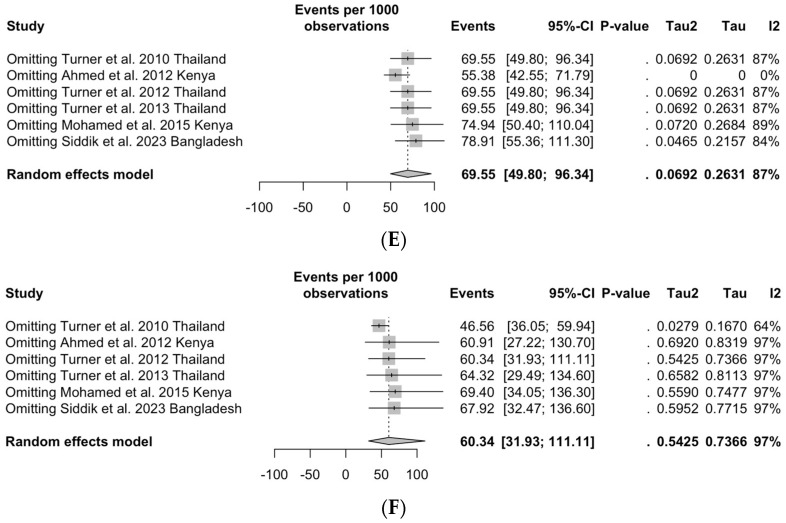
Forrest plots for sensitivity analysis on the pooled analyses on prevalence of RSV (**A**), influenza A (**B**), influenza B (**C**), human adenovirus (**D**), parainfluenza virus (**E**), and human metapneumovirus (**F**). Sensitivity analysis was performed by removing one study at time [5,7,45,47,48,77].

Sensitivity analysis was performed by removing one study at time, and both prevalence estimates and heterogeneity were calculated accordingly. Focusing on RSV, the removal of studies from Thailand reduced the pooled prevalence estimates to 121.42 per 1000 samples (95% CI 54.37 to 249.35), 104.6 (95% CI 53.96 to 193.07), and 112.38 (95% CI 52.66 to 233.83) (Figure A7A). On the contrary, the removal of Turner et al. 2010 [45] as well as of the study from Mohamed et al. [77] from Kenya substantially affected the estimates for influenza (95.54 per 1000 samples, 95% CI 67.78 to 125.13; 102.42 per 1000 samples, 95% CI 62.61 to 163.13, respectively; Annex Figure A7B). Focusing on the estimates for HAdV, the removal of Siddik et al. [47] led to an increased estimate (173.34 per 1000 samples, 95% CI 131.39 to 227.66) while omitting Ahmed et al. [48] was associated with a reduced pooled prevalence (114.70 per 1000 samples, 95% CI 67.94 to 187.17) (Figure A7D). Regarding hMPV, the removal of Turner et al., 2010 [45] led to a substantial reduction in pooled estimates (46.56 per 1000 samples, 95% CI 36.05 to 59.94) (Figure A7F). As highly expected, the removal of the study from Turner et al., 2010 [45] led to a stark increase in the estimates for influenza B (33.94 per 1000 samples, 95% CI 18.20 to 62.42) (Figure A7C).

When dealing with residual heterogeneity, sensitivity analysis did not report any substantial change. In fact, only estimates on hMPV were affected by the removal of the single study by Turner et al., 2010 [45], with I^2^ estimates dropping to 64% from the baseline 96.6% of the pooled sample.

### 3.7. Publication Bias and Small Study Bias

**Figure 7:**
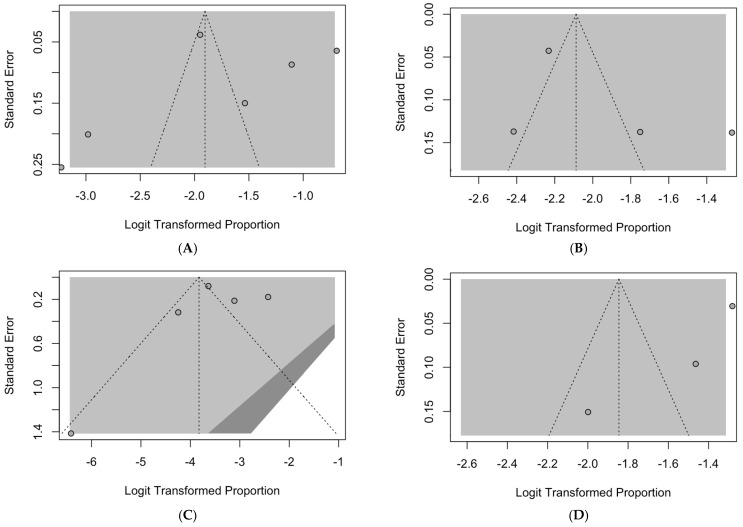

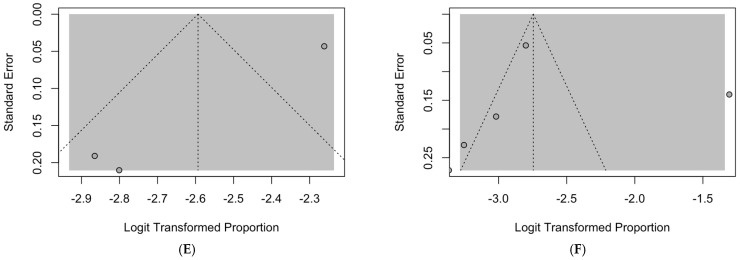
Funnel plots for estimates on RSV (**A**), influenza A (**B**), influenza B (**C**), human adenovirus (**D**), parainfluenza virus (**E**), and human metapneumovirus (**F**) [5,7,45,47,48,77].

To ascertain the residual publication bias, we initially calculated funnel plots on the prevalence estimates (see Figure 7).

In funnel plots, the sample size is plotted against the reported effect size (in this case, the pooled prevalence estimates). As the size of the sample increases, individual estimates of the effect should converge around the “true” estimate of that specific effect [63,66,73]. Taking into preliminary account that pooled analyses were are affected by a very limited number of collected series, requiring a cautious appraisal of visual inspection, all funnel plots were affected by substantial asymmetry, particularly the analyses on RAV (Figure 7A), HAdV (Figure 7C), and hMPV (Figure 7F). As a consequence, we cannot rule out that the meta-analysis summaries underestimated the prevalence rates for assessed respiratory pathogens.

**Figure A8:**
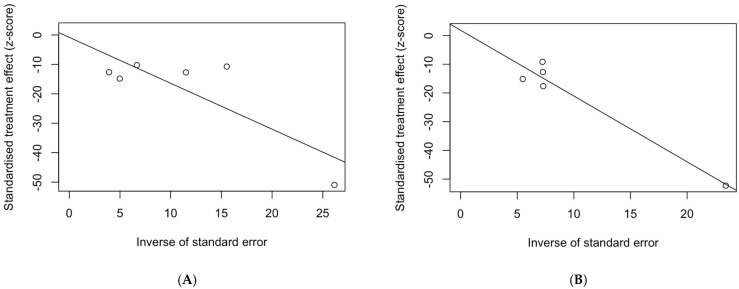

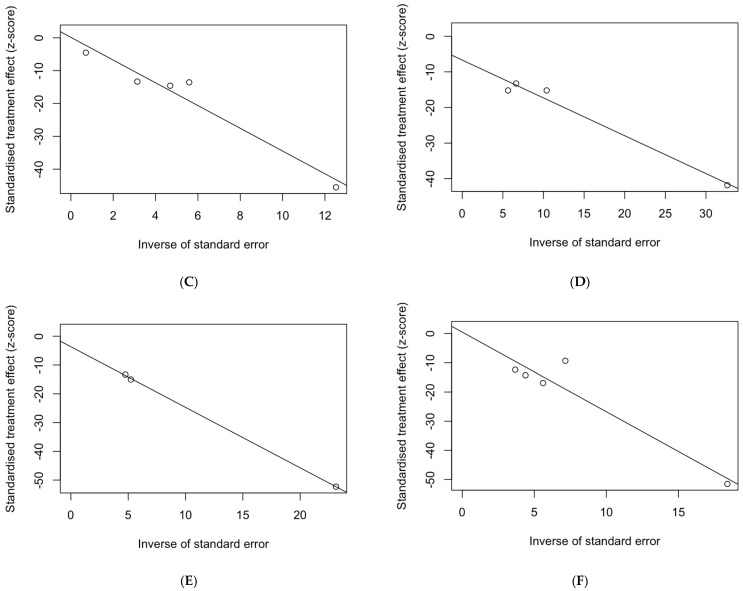
Radial plots for estimates on RSV (**A**), influenza A (**B**), influenza B (**C**), human adenovirus (**D**), parainfluenza virus (**E**), and human metapneumovirus (**F**) [5,7,45,47,48,77].

Radial plots have been similarly calculated (Appendix A, Figure A8), and their analysis through Egger’s test is summarized in Table 6. The reduced number of included samples suggests a very cautious appraisal of visual inspection given the uneven distribution of estimates on the two sides of the regression line.

Although visual inspection suggested a certain publication bias, analysis using Egger’s test stressed some residual bias only for HAdV (t = −3.24, *p* = 0.083) and PIFV (t = −3.64, *p* = 0.087), and both estimates were affected by a vey reduced number of included series (4 for HAdV, 3 for PIFV).

## 4. Discussion

### 4.1. Summary of Key Findings

In the present systematic review and meta-analysis, we were able to collect evidence from six studies (timeframe spanning from 2009 to 2020), including a total of 9319 samples. The six studies were from three areas, namely: Thailand [5,7,45], Bangladesh [47], and Kenya [48,77], and the results were reasonably affected by some degree of overlapping. However, a crude prevalence of RSV positive status was estimated at 15.18%, with individual rates of 3.83% for Bangladesh [47], 12.18% for the studies based on Thailand [5,7,45], and 30.01% for the studies from Kenyan refugee camps [48,77]. In other words, the risk estimates for RSV positive status was substantially greater in the studies from Thailand (RR 2.512, 95% CI 1.723 to 3.686) and Kenya (RR 6.209, 95% CI 4.239 to 9.093) than in the report from Bangladesh. Collected studies provided estimates for other respiratory pathogens, including influenza A (8234 total samples, prevalence of 10.03%), influenza B (8234 total samples, prevalence 2.88%), HAdV (7929 samples, prevalence of 19.90%), PIFV (7211 samples, prevalence of 8.92%), and hMPV (8234 samples, prevalence 5.96%). Using influenza A as a reference group, we identified a substantially increased risk for positive status regarding RSV (RR 1.514, 95% CI 1.396 to 1.641) and HAdV (RR 1.984, 95% CI 1.834 to 2.146), while all other pathogens were associated with lower risk (influenza B, RR 0.276, 95% CI 0.239 to 0.319; PIFV, RR 0.889, 95% CI 0.806 to 0.981; hMPV, RR 0.594, 95% CI 0.534 to 0.662). Interestingly enough, a positive RSV status was more frequently reported in cases of SARI and pneumonia (18.22%) than among collected ILIs (8.53%, RR 2.136, 95% CI 1.839 to 2.480). A similar proportion was found among cases of HAdV (21.89% vs. 18.76%, RR 1.167, 95% CI 1.052 to 1.259), while influenza B was less frequently associated with SARI (2.32%) than with ILIs (3.64%; RR 0.637, 95% CI 0.482 to 0.842). Eventually, a pooled estimate of 129.704 per 1000 samples (95% CI 66.393 to 237.986) was calculated for RSV, compared to 136.398 per 1000 samples (95% CI 84.510 to 212.741) for HAdV, 110.287 per 1000 samples (95% CI 73.186 to 162.889) for influenza A, 69.553 per 1000 samples (95% CI 49.802 to 96.343) for PIFV, 60.338 per 1000 samples (95% CI 31.933 to 111.109) for hMPV, and eventually 21.351 per 1000 samples (95% CI 7.319 to 60.639) for influenza B. All estimates were affected by substantial heterogeneity, particularly those for RSV (I^2^, 98.8%, 95% CI 98.3 to 99.1), while a more limited publication bias was identified. Finally, sensitivity analysis suggests that the removal of individual studies would not affect the heterogeneity of pooled analyses, particularly for RSV.

### 4.2. Generalizability

**Figure A9:**
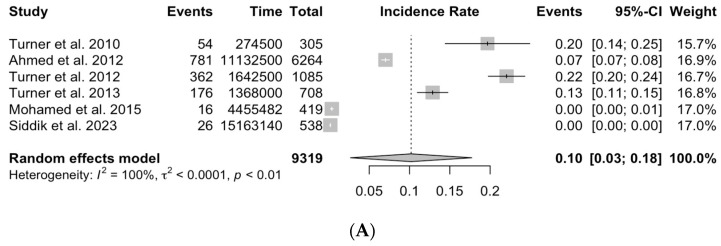

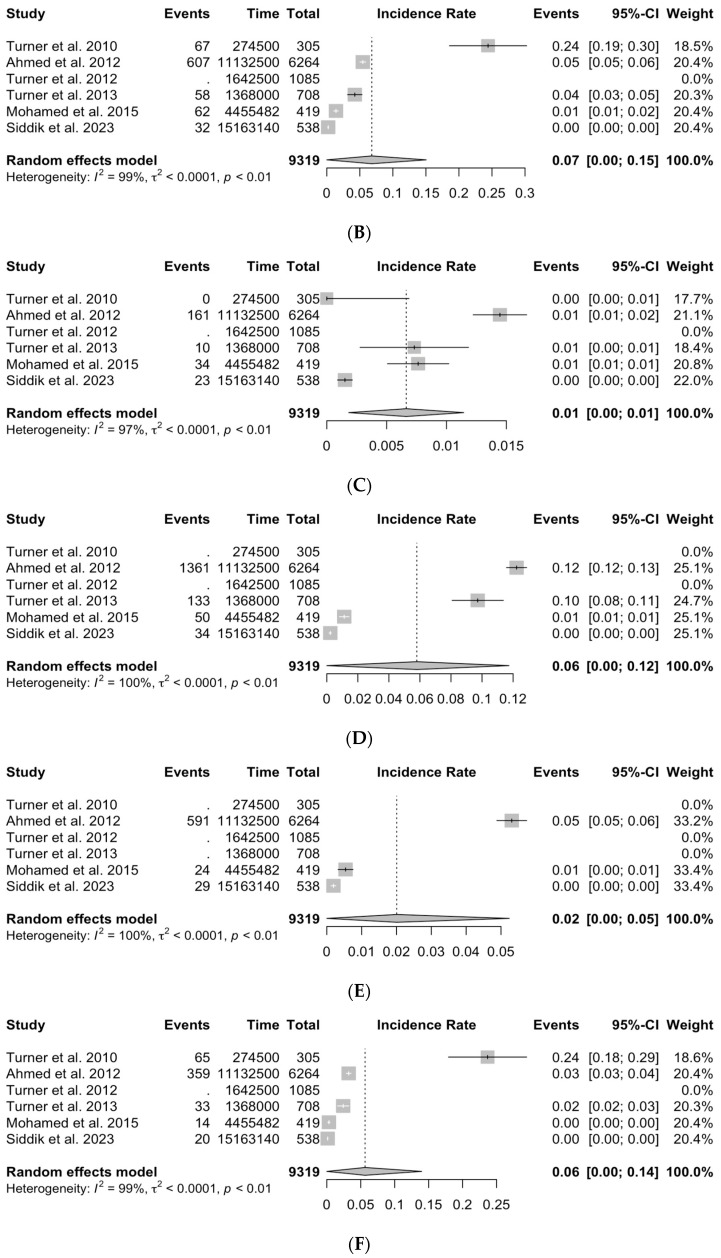
Forrest plots for incidence rates of RSV (**A**), influenza A (**B**), influenza B (**C**), human adenovirus (**D**), parainfluenza virus (**E**), and human metapneumovirus (**F**) infections. Data are reported in events per 1000 person-months [5,7,45,47,48,77].

A key issue for the appraisal of respiratory pathogens, particularly RSV, is represented by their seasonal trend. For example, in the Northern Hemisphere, RSV and influenza usually co-circulate during the winter season. Outbreaks start at the end of the calendar year (i.e., November or December), reaching their peak between January and February, with lower rates during the months of March and April, and even lower rates of new infections during the warm season [79,80,81,82]. On the contrary, peri-equatorial countries characterized by higher rainfall and humid weather may exhibit high rates throughout the calendar year or incidence peaks clustered within the rainy season [27,83,84]. Therefore, studies focusing on a reduced timeframe may result in either over- or underestimation of incidence rates in accordance with having or having not included the months characterized by highest circulation of the sampled pathogens (Figure A9). On the contrary, studies spanning the whole calendar year may be far more effective in assessing the actual activity of respiratory pathogens. Not coincidentally, the study by Turner et al. [45] spanning the summer season of 2009 was associated with far lower rates for RSV than those reported from the very same study group in two follow-up studies performed from 01 November 2007 to 31 October 2010 [5] and from 01 April 2009 to 30 September 2011 [7] (RSV prevalence 17.76% vs. 33.36% and 24.86%, respectively).

Another issue that could affect the generalizability of our results is represented by the sampling strategy. In fact, none of the retrieved studies performed a systematic sampling of all individuals hosted in the refugee camps. On the contrary, all reports collected the specimens from cases whose symptoms were consistent with an underlying respiratory tract infection, ranging from all incident cases of ARIs [47] to all cases of ILIs and/or pneumonia [45], all cases of pneumonia [5,7], and all cases of ILIs or SARIs [48,77]. In fact, ILIs represent an improper proxy for RSV infections, the clinical feature of which more properly fit SARI and LRTI definitions, which include bronchiolitis and pneumonia [4,48]. Therefore, studies encompassing a larger share of ILIs such as the reports from Turner et al. [45], Ahmed et al. [48], and Mohamed et al. [77] may in turn provide an underestimation of the actual RSV prevalence, with an oversampling of other respiratory pathogens. In our review, the studies with higher rates of ILI were actually characterized by high or even very high rates for influenza A (22.04%, 9.69%, and 14.80%), and HAdV (21.73% and 11.93% in the studies from Ahmed et al. [48] and Mohamed et al., [77], respectively), while the studies focusing on pneumonia [5,7] were characterized by the highest estimates for RSV (from 24.86% to 33.36%).

Sampling strategy may be also affected by characteristics of the refugee camps. For example, the refugee camp of Maela included around 45,000 individuals at its peak, the total population of the refugee camps from Kenya ranged from around 122,000 to over 300,000 inmates, and the Cox’s Bazar substantially exceeded 500,000 individuals at the time of the study from Siddik et al. [47]. According to the figures provided by the UNCHR, in 2011, the Maela camp (total surface of 1.84 square km) had a population density of around 24,450/km^2^, with 52% of refugees above 18 years, 33.5% between 5 and 18, and 14% less than 5 years. Despite its larger surface (13 square km) Cox’s Bazar is not only characterized by substantially higher population density (>46,000/km^2^), but the share of refugees aged above 18 years does not exceed 42%, with 37% between 5 and 18 years, and 14% less than 5 years. Moreover, refugees from Cox’s Bazar are more frequently from larger household, as 40% of families have a size of 4 to 5 persons, but 24% exceed 6 persons. Data on the Hagadera camps and the area of Daadab are far more uncertain, as a large share of sheltered individuals are unregistered. Still, available figures hint toward a similarly high share of individuals of a pediatric age (52% 0 to 17 years). As the circulation of respiratory pathogens is directly affected by the size and characteristics of the household, more densely populated camps, characterized by the highest shares of children and adolescents, should be affected by highest likelihood of infection. Quite unexpectedly, the study from Cox’s Bazar was characterized by relatively low rates for all sampled pathogens, but these results could be explained through the sampling strategy. Although the authors reportedly sampled all incident ARI cases with a well-defined clinical definition, how patients were actually enrolled remains unspecified. In other words, again, we cannot rule out the oversampling of cases where the underlying pathogen was represented by a bacterial pathogen, as suggested by the very high detection rate for *S. pneumoniae* (80.86%), *S. aureus* (21.84%), and *H. influenzae* (around 15%).

### 4.3. Limits and Implications for Future Studies

Our study is reasonably affected by some significant limits that should be considered.

First, we must acknowledge the limited number of studies that we were able to retrieve. In fact, we deliberately applied a strict search strategy to guarantee the good or very good quality of retrieved studies and to improve the overall reliability of the results. Still, our research strategy resulted in a small number of studies being ultimately collected and included in the meta-analysis. Moreover, the results were focused on studies set in only three countries (Bangladesh, Kenya and Thailand), and no other information was retrieved from refugee camps in other geographical areas [8] or from other refugee camps in the same areas. At the same time, the overall sample included less than 10,000 individuals and 9319 samples from an overwhelmingly larger population (approximately exceeding 1 million people).

Second, individual features of refugees are strikingly heterogenous across the various settings because of their demographics and underlying risk factors for adults (e.g., proportion and severity of substance abuse, smoking history, exposure to respiratory toxicants) and children (e.g., nutritional status, potential reliance on healthcare interventions from local health and governmental authorities, etc.) [8]. Unfortunately, such data were irregularly provided, and our understanding of the epidemiology of RSV and other respiratory infections across refugee camps could have been therefore flawed. In this regard, data on housing are usually not provided, particularly information on the use of biomass combustion for heating food and water. Consolidated evidence did repetitively stress how particulate matter resulting from the burning of biomasses represents effective risk factors for respiratory infections [85,86], including RSV [87].

Third, studies were conducted in a very broad timeframe, with a pluriannual hiatus between the reports from Thailand [5,7,45] and Kenya [48,77] on the one hand and the single report from Cox’s Bazar on the other hand [47]. Therefore, the eventual estimates may have been affected not only by inconsistency in the epidemiology of respiratory pathogens across the assessed timeframe [88,89], but also by available diagnostic options [88]. In this regard, it is important to stress that all studies were performed well before the inception of the SARS-CoV-2 pandemic. Therefore, the epidemiology of sampled respiratory viruses was not affected by local countermeasures, and more precisely by non-pharmaceutical interventions [90,91]. Even the study from Siddik et al. [47] encompassed a timeframe excluding the first months of the SARS-CoV-2 pandemic. At the same time, given that these results were collected in settings unaffected by COVID-19 and the resulting disruption of the seasonal epidemiology of influenza (A/B) and RSV, the representativity of collected results in the post-pandemic settings could be reasonably questioned.

Another factor to be addressed is the average size and composition of the households from assessed refugee camps, which are heterogenous across the sampled refugee camps. According to available estimates from the UNHCR, by 31 January 2023, all of the refugees from Myanmar to Bangladesh, including Cox’s Bazar, included around 954,707 people from 197,303 households [92], representing an average of 4.8 subjects per household. Among sheltered individuals, 44.6% were aged 18 to 59 years, while children aged 4 years or less accounted to 16.1%. On the contrary, the figures for all of the refugee camps from Kenya hint at a lower proportion of children 0 to 4 years (12.8%), with a comparable share of adults 18 to 59 years (45.0%). Focusing on Dadaab, by 29 February 2024, the UNHCR reported a total of 381,217 refugees from 76,163 households, representing an average of 5.0 individuals per household [93]. In fact, considerable evidence suggests that RSV differs from other communicable pathogens, such pertussis, as seasonal outbreaks are maintained by “peers” (i.e., siblings and children of comparable age) rather than by older individuals with waning immunity [25,94]. Hence, even transitory heterogeneities in household size and daily interactions with individuals potentially at risk eventually result in heterogenous rates for RSV infections.

In other words, from a short-term perspective, the evidence we were able to recollect could be therefore only limitedly applicable in the current context of the ongoing refugee crisis, particularly when dealing with the Ukraine migratory crisis in Eastern and Central Europe [8]. On the other hand, from a long-term perspective, the potential differences in the size and composition of refugee camps urge for the appropriate tailoring of preventive measures. For instance, the American Centers for Disease Control and Prevention recently recommended RSV vaccines for adults 60 years of age and older at highest risk for severe RSV disease [58,95]. Similar recommendations have been issued for pregnant people to protect their babies from severe RSV disease by means of a single dose of bivalent RSVpreF vaccine during weeks 32 through 36 of pregnancy during September through January [59,96]. This strategy may be considered as a complimentary one for other interventions, including the delivery of mAb (e.g., Palivizumab or Nirsevimab) to either high-risk newborns [97] or more extensive population groups [98]. However, all options should be accurately taken into account in accordance with underlying settings and available resources.

## 5. Conclusions

RSV was characterized as a likely occurrence in refugee camps, with noticeable disease burden, even compared to other respiratory pathogens such as influenza A, hMPV, and HAdV. Collected data were reasonably affected by characteristics of factors such as heterogeneities in sample size, specificities of the refugee camps, and evolving epidemiological features of respiratory virus infections. However, despite the unfavorable settings and the limited resources usually associated with refugee camps, the availability of effective immunizations against RSV, including the maternal vaccination strategy, stresses the potential public health and social value of preventive interventions for RSV in these high-risk settings.

## Figures and Tables

**Table 1:** Summary of retrieved studies on RSV and respiratory viral infections among refugees and asylum seekers from refugee camps (Notes: ILI = influenza like illness; SARI = severe acute respiratory infection) [5,7,45,47,48,77].

Study	Country	Timeframe	Settings	Total Population of Refugees	Sampling Strategy	Patients (n.)	Age	Samples (N.)	Males (n./N., %)	ILI (n./N., %)	SARI/Pneumonia (n./N., %)
Turner et al., 2010 [45]	Thailand	01 May 2009 to 31 October 2009	Refugee camp, Maela	45,000 (approximately)	All incident cases of ILI or pneumonia	324	ILI: 1.4 y.o. (range 0.4–1.0) Pneumonia: 2.0 y.o. (range 0.1.–68)	305	165 (54.10%)	71 (23.28%)	234 (76.72%)
Ahmed et al., 2012 [48]	Kenya	01 September 2007 to 31 August 2010	Refugee camp, Dadaab areas; Hagadera, Ifo and Dagahaley camps	305,000 (approximately)	All incident cases of ILI or SARI	6647	<1 y.o. 2992 (45.01%) 1 to <2 y.o.: 1379 (20.75%) 2 to <5 y.o. 1467 (22.07%) ≥5 y.o. 1126 (16.94%)	6264	2870 (45.82%)	1815 (28.98%)	4449 (71.02%)
Turner et al., 2012 [5]	Thailand	01 November 2007 to 31 October 2010	Refugee camp, Maela	45,000 (approximately)	All incident cases of pneumonia	955	N.A.	1085	NA	0	955 (100%)
Turner et al., 2013 [7]	Thailand	01 April 2009 to 30 September 2011	Refugee camp, Maela	45,000 (approximately)	All incident cases of pneumonia	698	<1 y.o. 289 (40.82%) 1 to 4 y.o. 351 (49.58%) ≥5 y.o. 68 (9.6%)	708	404 (57.06%)	0	698 (100%)
Mohamed et al., 2015 [77]	Kenya	01 January 2010 to 31 December 2012	Refugee camp, Dadaab areas; Hagadera	122,068	All incident cases of ILI or SARI	471	5 to 14 y.o. 345 (73.25%) 15 to 24 y.o. 54 (11.46%) 25 to 54 y.o. 67 (14.23%) ≥65 y.o. 5 (1.06%)	419	259 (54.99%) *	200 (47.73%)	169 (40.33%)
Siddik et al., 2023 [47]	Bangladesh	01 March 2018 to 30 March 2020	Cox’s Bazar	Not reported (598,545)	All incident cases of ARI	538	≤5 y.o. 320 (59.48%) >5 y.o. 218 (40.52%)	538	291 (54.09%)	0	538 (100%)

* Proportion calculated over the number of potentially sampled refugees.

**Table 2:** Prevalence of positive samples for viral respiratory infections among refugees and asylum seekers (Note: RSV = respiratory syncytial virus; Flu. = influenza; HAdV = human adenovirus; PIFV = parainfluenza virus; hMPV = human metapneumovirus) [5,7,45,47,48,77].

Study	Total Samples (N./TOT, %)	RSV (n./N., %)	Flu. A (n./N., %)	Flu. B (n./N., %)	HadV (n./N., %)	PIFV (n./N., %)	hMPV (n./N, %)
Turner et al., 2010 [45]	305 (3.27%)	54 (17.76%)	67 (22.04%)	0 (-)	-	-	65 (21.38%)
Ahmed et al., 2012 [48]	6254 (67.22%)	781 (12.47%)	607 (9.69%)	161 (2.57%)	1361 (21.73%)	591 (9.43%)	359 (5.73%)
Turner et al., 2012 [5]	1085 (11.64%)	362 (33.36%)	-	-	-	-	-
Turner et al., 2013 [7]	708 (7.60%)	176 (24.86%)	58 (8.19%)	10 (1.41%)	133 (18.79%)	-	33 (4.66%)
Mohamed et al., 2015 [77]	419 (4.50%)	16 (3.82%)	62 (14.80%)	34 (8.11%)	50 (11.93%)	24 (5.73%)	14 (3.34%)
Siddik et al., 2023 [47]	538 (5.77%)	26 (4.83%)	32 (5.95%)	23 (4.28%)	34 (6.32%)	29 (5.39%)	20 (3.72%)
TOTAL	9319	1415/9319 (15.18%)	826/8234 (10.03%)	228/8234 (2.88%)	1578/7929 (19.90%)	644/7221 (8.92%)	491/8234 (5.96%)

**Table 3:** Prevalence of positive samples for viral respiratory infections among refugees and asylum seekers by geographical distribution of included studies (Note: RSV = respiratory syncytial virus; Flu. = influenza; HAdV = human adenovirus; PIFV = parainfluenza virus; hMPV = human metapneumovirus) [5,7,45,47,48,77].

Country	RSV		Flu.A		Flu.B		hAdV		PIFV		hMPV	
	Tot.	Pos. (%)	Tot.	Pos. (%)	Tot.	Pos. (%)	Tot.	Pos. (%)	Tot.	Pos. (%)	Tot.	Pos. (%)
Bangladesh [47]	538	26 (4.83%)	538	32 (5.95%)	538	23 (4.28%)	538	34 (6.32%)	538	29 (5.39%)	538	20 (3.72%)
Thailand [5,7,45]	6988	851 (12.18%)	6988	736 (10.53%)	6988	195 (2.79%)	6683	1411 (21.11%)	6683	615 (9.20%)	6988	438 (6.27%)
Kenya [48,77]	1793	538 (30.01%)	708	58 (8.19%)	708	58 (8.19%)	708	133 (18.79%)	-	-	708	33 (4.66%)

**Table 4:** Detailed reporting of the risk of bias (ROB) estimates for observational studies [70,71]. Analyses were performed according to the National Toxicology Program (NTP)’s Office of Health Assessment and Translation (OHAT) handbook and respective risk of bias (ROB) tool. Note: D1: possibility of selection bias; D2: exposure assessment; D3: outcome assessment; D4: confounding factors; D5: reporting bias; D6: other bias; ☹: probably high; ☺: probably low; ☺☺: definitively low.

Study	D1	D2	D3	D4	D5	D6
Turner et al., 2010 [45]	☺	☺	☺	☺	☺	☺
Ahmed et al., 2012 [48]	☺	☺	☺☺	☺☺	☹	☺
Turner et al., 2012 [5]	☺	☹	☺	☺	☹	☺
Turner et al., 2013 [7]	☺☺	☺	☺	☺☺	☺	☺
Mohamed et al., 2015 [77]	☺	☺	☺☺	☺☺	☺	☺
Siddik et al., 2023 [47]	☹	☹	☺☺	☺☺	☺	☺

**Table 5:** Summary of pooled prevalence estimates for respiratory viruses included in the analyses. Note: RSV = respiratory syncytial virus; HAdV = human adenovirus; PIFV = parainfluenza virus; hMPV = human metapneumovirus.

Pathogen	Pooled Prevalence (N./1000 Samples, 95% CI)	τ^2^	(I^2^; 95% CI)	Q	*p* Value
RSV	129.704 (66.393; 237.986)	0.832	98.8% (98.3 to 99.1)	402.35 (df = 5)	<0.001
Influenza A	110.287 (73.186; 162.889)	0.247	94.1% (89.1 to 96.8)	67.70 (df = 4)	<0.001
Influenza B	21.351 (7.319; 60.639)	1.265	91.5% (83.1 to 95.7)	46.85 (df = 4)	<0.001
HAdV	136.398 (84.510; 212.741)	0.284	96.4% (93.3 to 98.0)	37.58 (df = 3)	<0.001
PIFV	69.553 (49.802; 96.343)	0.069	86.8% (62.2 to 95.4)	15.16 (df = 2)	0.001
hMPV	60.338 (31.933; 111.109)	0.543	96.6% (94.3 to 98.0)	117.71 (df = 4)	<0.001

**Table 6:** Summary of Egger’s test on the residual publication bias on studies for respiratory viruses included in the analyses. Note: RSV = respiratory syncytial virus; HAdV = human adenovirus; PIFV = parainfluenza virus; hMPV = human metapneumovirus.

Pathogen	t	df	*p* Value	Bias (SE)	Intercept (SE)
RSV	−0.11	4	0.916	−0.822 (7.340)	−1.560 (0.532)
Influenza A	0.49	3	0.655	1.828 (3.716)	−2.291 (0.306)
Influenza B	0.05	3	0.964	0.154 (3.099)	−3.464 (0.467)
HAdV	−3.24	2	0.083	−6.688 (2.061)	−1.063 (0.117)
PIFV	−3.64	1	0.078	−3.644 (0.448)	−2.105 (0.032)
hMPV	0.10	3	0.926	0.495 (4.929)	−2.756 (0.517)

## Data Availability

Full data are available on request to the principal investigator.

## Supplementary Materials

The following supporting information can be downloaded at: https://www.mdpi.com/article/10.3390/epidemiologia5020016/s1: Table S1: PRISMA checklist.

## Appendix A

**Table A1:** Search strategy and number of retrieved entries.

Repository	Search Strategy	Number of Entries
PubMed	(“respiratory infection*” OR “Respiratory Syncytial Virus, Human”[Mesh] OR “Respiratory Syncytial Viruses”[Mesh] OR “Respiratory Syncytial Virus Infections”[Mesh]) AND (“Refugee Camps”[Mesh] OR “Refugees”[Mesh] OR refugee* OR asylum)	86 (12.25%)
EMBASE	(“human respiratory syncytial virus” OR “respiratory infections” OR “respiratory syncytial virus infection” OR “pneumovirus” OR “pneumovirus infection”) AND (“refugee” OR “refugee camp” OR “asylum seeker” OR “asylum seeker center”)	77 (10.97%)
Scopus	(RSV OR “respiratory syncytial virus” OR “respiratory infection*”) AND (refugee* OR “refugee camp*” OR “displaced” OR “asylum seeker*”)	136 (19.37%)
medRxiv	“respiratory syncytial virus” OR RSV OR “respiratory infection”) AND (“refugee” OR “asylum seeker”)”	403 (57.41%)
TOTAL		702
